# Identifying and prioritizing do-not-do recommendations in Dutch primary care

**DOI:** 10.1186/s12875-022-01713-y

**Published:** 2022-06-03

**Authors:** Simone A. van Dulmen, Ngoc Hue Tran, Tjerk Wiersma, Eva W. Verkerk, Jasmine CL Messaoudi, Jako S. Burgers, Rudolf B. Kool

**Affiliations:** 1 Radboud university medical center, Radboud Institute for Health Science, IQ healthcare, PO Box 9101 (160), 6500 HB Nijmegen, the Netherlands; 2 Dutch College of General Practitioners, Mercatorlaan 1200, 3528 BL Utrecht, the Netherlands; 3 Department Family Medicine, Care and Public Health Research Institute, Peter Debyeplein 1, 6229 HA Maastricht, the Netherlands

**Keywords:** Family practice, General practitioners, Netherlands, Primary health care, Clinical guidelines, Low-value care, De-implementation

## Abstract

**Background:**

Low-value care provides minimal or no benefit for the patient, wastes resources, and can cause harm. Explicit do-not-do recommendations in clinical guidelines are a first step in reducing low-value care. The aim of this study was to identify and prioritize do-not-do recommendations in general practice guidelines with priority for implementation.

**Methods:**

We used a mixed method design in Dutch primary care. First, we identified do-not-do recommendations through a systematic assessment of 92 Dutch guidelines for general practitioners (GPs), resulting in 385 do-not-do recommendations. Second, we selected 146 recommendations addressing high prevalent conditions. Third, a random sample of 5000 Dutch GPs was invited for an online survey to prioritize recommendations based on the prevalence of the condition and low-value care practice, potential harm, and potential cost reduction on a scale from 1 to 5/6. Total scores could range from 4 to 22. Recommendations with a median score > 12 were included. In total, 440 GPs completed the survey.

**Results:**

The selection process led to 30 prioritised recommendations. These covered drug treatments (*n* = 12), diagnostics (*n* = 10), referral to other healthcare professions (*n* = 5), and non-drug treatment (*n* = 3).

**Conclusion:**

Dutch clinical guidelines include many do-not-do recommendations that are perceived as highly relevant by the GPs. The list of 30 high-priority do-not-do recommendations can be used to raise awareness of low-value care among GPs. As the recommendations are supported with the latest evidence from international studies, primary healthcare professionals and policy makers worldwide can use the list for further validating the list in their local context and designing strategies to reduce low-value care.

## Background

In all sectors of medical practice healthcare professionals provide low-value care procedures and treatments [1]. Low-value care can be defined as care that provides minimal or no benefit, considering harms, costs, alternatives, and patient preferences [2]. De-implementation of low-value care can improve the quality of care and reduce costs [3]. Various initiatives have been started to identify low-value care practices. Examples are the ‘Choosing Wisely’ campaign in various countries, ‘Smarter Medicine’ in Switzerland, and a list developed by the United Kingdom’s National Institute for Health and Care Excellence (NICE) [4–6]. Multiple lists with do-not-do recommendations have been compiled [7–10]. These recommendations advise clinicians to refrain from diagnostics and treatments that are proven of low value. These lists are mainly focused on hospital care and include only a few numbers of do-not-do recommendations for primary care such as in Switzerland, Canada and the United States [8, 11, 12].

In the Netherlands, medical specialists [10] and nurses [13] have created lists containing respectively 1366 and 66 low-value care practices. With these lists the awareness of low-value care practice can be increased as a first step in de-implementation [10, 14]. Dutch GPs have an important role as gatekeeper to hospital- and specialist care. They are the first healthcare providers for patients with a wide range of conditions. All Dutch citizens are registered with a GP and 78% consult their GP in a year time [15]. GPs can therefore have a significant impact in reducing low-value care to prevent needless harm to patients and to reduce healthcare costs. Well-known examples of low-value care practices in primary care are imaging in patients with non-specific low back pain when red flags are not present, [16] and prescribing antibiotics in patients with non-severe pharyngotonsillitis [17]. Dutch GPs revealed that 99% of them are aware that low-value care is provided in primary care and two-thirds (67%) responded that it occurs regularly or often [18]. Unnecessary medication (27%), laboratory tests (25%), such as vitamin and PSA tests, and imaging (18%) were most frequently mentioned low-value care practices [18].

However, it is still challenging for GPs to refrain from providing low-value care. Increased cost-consciousness and awareness of low-value care among GP’s is associated with providing less low-value practices [19]. Therefore, a list of do-not-do recommendations in general practice can guide de-implementation activities. The aim of this study is to select do-not-do recommendations with high priority for de-implementation based on the opinion of GPs on prevalence, harm to patients, and potential cost reduction.

## Methods

### Design and setting

The Dutch College of General Practitioners has a long-standing guideline program since 1989. In 2018, 134 guidelines were available. We used the complete set of guidelines to identify recommendations stating that specific interventions should be avoided. We aimed to select and to prioritise do-not-do recommendations in a stepwise approach in three steps [7].

- Step 1: Identifying do-not-do recommendations

We started with all the 134 guidelines that were available in the database of the Dutch College of General Practitioners in September 2018 and endorsed by the Dutch College of GPs. Fifteen guidelines were published before 2008 and 27 guidelines were being revised or planned for revision in 2018 or in 2019. These guidelines were excluded to prevent the inclusion of recommendations that are not applicable to current practice. In total 92 guidelines were screened for do-not-do recommendations.

A do-not-do recommendation was defined as a recommendation that instructs the GP not to offer specific interventions. We first randomly selected 5 practice guidelines that were analysed by three researchers (JM, LW and EV) to identify do-not-do recommendations. The researchers compared their results and consulted three other researchers (SAvD, RBK and TW) until agreement was reached. Subsequently, we repeated this process for 5 randomly selected guidelines to further refine the inclusion and the exclusion criteria. To evaluate the agreement, another ten guidelines were independently screened to determine the inter-rater reliability. Inter-rater reliability was analysed by calculating Fleiss’ Kappa (k) for multiple raters [20]. Using the method described, the remaining 72 guidelines were screened by one researcher (JM). Difficulties in interpretation were discussed with a second researcher (SAvD or RBK) and when necessary, with a third researcher (TW) until consensus was reached.

We included recommendations that applied to practice usually performed by GPs. Elective low-value procedures, such as fundoscopy for diagnosing eye problems, were excluded, as they are only performed by few (specialised) GPs in the Netherlands. Furthermore, recommendations containing only an advice for the patient or stating the obvious or a well-known contra-indication or interaction, were excluded after discussion with the second or third researcher. For example, ‘Stop using nifedipine for Raynaud’s syndrome if the treatment is ineffective and/or causes undesirable side effects’ (Guideline Raynaud’s syndrome, 2018). Recommendations just stating well-known contra-indications or interactions were excluded. For example, ’Do not prescribe NSAID (or acetylsalicylic acid) to patients who had an anaphylactic reaction to NSAID in the past’ (Guideline Pain, 2018). We combined similar recommendations found in one guideline and removed duplicates found in multiple guidelines.

- Step 2: Selecting do-not-do recommendations

In the second step, we reduced the number of recommendations in order to have an appropriate number feasible for step three. Recommendations were divided among two researchers (NHT and TW, who is a practicing GP and in charge of the College of General Practioners) and screened independently. Recommendations regarding low-prevalent diagnostics, treatments, procedures and referrals were excluded. The prevalence was estimated based on NHT and TW’s experienced frequency of recommendation-related visits to the GP practice. Symptoms or illnesses that GPs encounter approximately less than once a month were excluded. Any uncertainties were discussed with a second and third researcher (RBK and SAvD) until consensus was reached. We categorised the recommendations in diagnostics, drug treatments, non-drug treatments, referrals, and miscellaneous.

- Step 3: Prioritizing do-not-do recommendations by GPs

We used an online survey to assess the opinions of GPs in regard to four criteria of the recommendation based on previous research. [7, 21]: 1) prevalence of the condition, 2) prevalence of the low-value care practice, 3) potential harm to the patient, and 4) potential for cost reduction. The online survey was pilot tested by a team of eight researchers, GPs and medical students). After feedback and refinements, the do-not-do recommendations selected in step 2 were randomly distributed into five different online surveys designed in LimeSurvey Version 2.06+ to keep the length of the survey limited and thereby increase the response rate. Invitations for each online survey were sent by the office of the Dutch College of General Practitioners to 1000 GPs, using random sampling of the national database of their members. The majority of the Dutch GPs are member of the Dutch College of General Practitioners. That database consists of 12.766 GPs. Doctors in training for GP were excluded. We aimed for a response of a minimum of 100 GPs for each recommendation to be able to estimate the support for de-implementation of the low-value care practice. Considering the estimated duration of the online survey (20 minutes) the response rate was estimated at 10% based on previous experiences. We developed five surveys (in Dutch) and randomly assigned the GPs to one of the five surveys (1000 per survey). In total 5000 GPs were invited to evaluate the do-no-do recommendations.

Table 1 describes the questions and answering options used in the survey. We used a scale from 1 (never) to 6 (very often) for assessing the prevalence of the clinical condition (criterium 1) and the estimation of the prevalence of providing the mentioned low-value care practice by GPs (criterium 2) on a scale from 1 (never) to 6 (very often). The potential harm to the patient (criterium 3) was assessed on a scale from 1 (none) to 5 (major). The potential cost reduction (criterium 4) was assessed on a scale from 1 (don’t agree) to 5 (fully agree), and with an ‘I don’t know’ option. The latter answering option was excluded from the analyses. Non responders received one reminder to complete the online survey. The mean score with a standard deviation (SD) and the median with an interquartile range (IQR) were calculated for each criterium. Total scores could range from 4 to 22. We aimed to identify high priority recommendations, and a manageable number of recommendations to communicate to GPs and where GPs can choose from for potential future de-implementation strategies. Therefore, recommendations with a median score of 13 or higher were selected. The results were analysed with SPSS Statistics 25.

**Table 1:** Questions for survey general practitioners

Question	Answering options					
	1	2	3	4	5	6
**Prevalence of the symptoms/clinical picture**: How often do you see patients with these complaints in the general practice?	Never	Rarely	Sometimes	Regular	Frequently	Very often
**Prevalence of the low-value care practice:** How often do you deviate from this recommendation?	Never	Rarely	Sometimes	Regular	Frequently	Very often
**Potential harm to the patient**: What is the burden for the patient if you deviate from the recommendation (e.g. invasive examination, side effects, complications, time)?	None	Small	Medium	Large	Major	
**Potential for cost reduction**: By implementing this recommendation healthcare costs could be reduced	Don’t agree	Somewhat disagree	Neutral	Somewhat agree	Fully agree	Don’t know

## Results

**Fig. 1:**
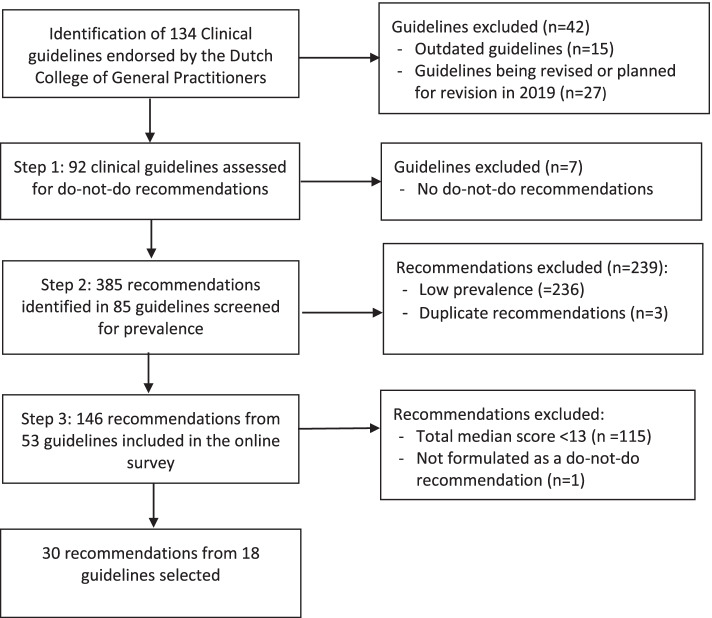
Flow diagram

Figure 1 shows the flow diagram of the identified guidelines and recommendations. In step 1, 92 guidelines were assessed for do-not-do recommendations. Seven guidelines did not include any do-not-do-recommendation. The calculated Fleiss Kappa for assessing inter-rater reliability was 0.715, indicating acceptable agreement [22].

In step 2, from the 385 do-not-do recommendations identified, 3 duplicate recommendations were removed and 236 recommendations were excluded based on low prevalence. This resulted in in 146 do-not-do recommendations included in the online survey. These recommendations were extracted from 53 guidelines with a range of 1 to 8 do-not-do-recommendations per guideline. The majority of the 146 recommendations (88%) concerned care that should not be provided at all, whereas others (12%) referred to care that could be offered with restraint. Of all recommendations, 49% covered drug treatment, 33% diagnostics, 8% non-drug treatment, 4% miscellaneous (e.g. follow-up appointments, combinations of diagnostics and drug treatment), 4% referral to specialist care and 2% covered referral to an another healthcare provider in primary care, such as a physiotherapist. We did not find a clear pattern between the year of publication and the number of recommendations. Guidelines with many do-not-do recommendations in step 2 were the guidelines ‘Lumbar radiculair syndrome’ (*n*=8), ‘Acute rhinosinusitis’ (*n*=7), ‘Pain’ (*n*=7), ‘Acute otitis media’ (*n*=7), and Thyroid disorders’ (*n*=7).

In total 440 GPs completed one out of the five online surveys. Four surveys contained 29 do-not-do recommendations and one survey contained 30 recommendations. Each recommendation was assessed by an average of 88 GPs (response rate 8.8%), with a range of 67–108 GPs. The mean age of the GPs was 46.7 years (SD 10.1; range 28-71 years), which is representative for the Dutch population of GPs [23]. The number of respondents was representative with regard to the distribution of GPs per province [23]. Female GPs were over-represented in our study; 67% in our study compared to 58% of all Dutch GPs [24]. The mean percentages female GPs ranged from 63% - 75% between the five survey groups.

All do-not-do recommendations with a median score of 13 or higher were selected, resulting in a total of 30 recommendations from 18 guidelines (see Table 2). Five recommendations concerned low back pain from the guidelines ‘Lumbar radicular syndrome’ and ‘Non-specific low back pain’. Both guidelines recommend against imaging and prescription of benzodiazepines. Four recommendations were included from the guideline ‘Non-traumatic knee complaints’ and three recommendations were included from the guidelines ‘Red eye and eye trauma’ as well as ‘Acute otitis media’. The 30 recommendations covered 33% diagnostics (*n*=10) and 66% treatment interventions (*n*=20), including drug treatments (*n*=12), referral to other healthcare professions (*n*=5) and non-drug treatment (*n*=3).

**Table 2:** Assessment of do-not-do recommendations on prevalence of low-value care, potential harm and cost reduction

			Prevalence of condition (range 1–6)		Prevalence of the low-value care practice (range 1–6)		Potential harm to patient (range 1–5)		Potential cost reduction (range 1–5)		Total score (range 4–22)	
	Guideline	Recommendation	Mean (SD)	Median (IR)	Mean (SD)	Median (IR)	Mean (SD)	Median (IR)	Mean (SD)	Median (IR)	Mean	Median
**Head region**	Red Eye and eye trauma	An antibiotic for an infectious conjunctivitis due to a banal pathogen is not indicated, unless for a high risk patient or when the symptoms persist for longer than 2 weeks	4.4 (0.7)	4 (1)	3.5 (1.0)	4 (1)	2.2 (0.8)	2 (1)	4.3 (0.8)	4 (1)	14.3	14
		If there are no complications, also in patients with complaints for more than one week there is no need to treat patients with symptoms of conjunctivitis with antibiotics	4.1 (0.8)	4 (1)	3.4 (1.0)	3 (1)	2.1 (0.7)	2 (0)	3.9 (1.1)	4 (2)	13.4	13
		A local antibiotic is not indicated in traumatic eye-injury due to non-severe etching	3.5 (1.1)	4 (1)	3.2 (1.4)	3 (2)	1.9 (0.6)	2 (0)	3.4 (1.2)	4 (1)	12.0	13
	Sleep disorders	Anti-psychotics, such as quetiapine, and sedative anti-histaminic are not indicated as treatment for insomnia	3.9 (1.0)	4 (1)	2.1 (1.1)	2 (2)	2.58 (0.9)	3 (1)	3.6 (1.0)	4 (1)	12.2	13
	Acute rhinosinusitis	Oral corticosteroids are not recommended for patients with acute sinusitis	4.0 (1.0)	4 (0)	2.4 (1.0)	2 (1)	2.9(1.0)	3 (2)	4.09 (1.2)	4 (2)	13.3	13
	Acute otitis media	Do not prescribe decongestive nasal spray as treatment for acute otitis media in children	4.6 (0.9)	5 (1)	2.8 (1.4)	3 (3)	2.1 (0.7)	2 (0)	3.0 (1.3)	3 (2)	12.4	13
		Oral antibiotics are not indicated in children with acute otitis media	4.6 (0.9)	5 (1)	2.4 (0.7)	2 (1)	2.6 (0.8)	3 (1)	4.1 (0.9)	4 (1)	13.7	14
		Antihistaminics, intranasal decongestiva, mucolytica and intranasal corticosteroids are not recommended as treatment of otitis media with effusion	4.2 (0.9)	4 (1)	2.8 (1.3)	3 (2)	2.4 (0.8)	2 (1)	3.8 (1.2)	4 (2)	13.2	13
**Abdominal region**	Acute diarrhea	The GP should refrain from ordering feces analysis	3.9 (0.9)	4 (0)	2.1 (0.8)	2 (0)	2.0 (0.8)	2 (0)	4.5 (0.8)	5 (1)	12.5	13
	Thyroid disorders	Routine blood testing of the thyroid function in patients with a depression or anxiety disorder is not recommended	3.9 (0.9)	4 (1)	2.6 (1.2)	3 (1)	2.18 (0.7)	2 (1)	3.7 (1.8)	4 (1)	12.4	13
	Abdominal pain in children	Do not refer children with non-somatic abdominal pain to a pediatrician	3.8 (0.9)	4 (1)	2.8 (0.9)	3 (1)	3.1 (0.7)	3 (1)	4.3 (1.0)	5 (1)	13.9	15
		Diagnostic tests for celiac disease, H. pylori, food allergies and lactose-intolerance in children with abdominal pain, without somatic cause are not recommended	4.0 (0.9)	4 (1)	2.8 (0.9)	3 (1)	2.6 (0.9)	2 (1)	4.1 (1.2)	4 (2)	13.4	13
	Stomach disorders	In H. pylori-negative patients younger than 50 years, with persisting or recurring stomach complaints, but without red flags, gastroscopy is not recommended	3.7 (0.8)	4 (1)	2.7 (0.8)	3 (1)	3.32 (0.9)	3,5 (1)	4.4 (1.0)	5 (1)	14.1	15,5
		Prevent chronic use of anti-acid medication, without adequate indication	4.4 (0.9)	4 (1)	3.2 (0.9)	3 (1)	2.76 (0.7)	3 (1)	4.3 (0.9)	5 (1)	14.8	15
	Rectal blood loss	Do notroutinely repeat a coloscopy within less than 10 years in patients with a positive immune-chemical fecal occult blood test (iFOBT) and a negative coloscopy	2.8 (0.9)	3 (1)	1.9 (0.7)	2 (1)	3.4 (0.9)	4 (1)	4.4 (0.9)	5 (1)	12.5	14
**Musculoskeletal**	Epicondylitis	Referring to a physiotherapist or to a (orthopedic) surgeon for an epicondylitis are not recommended	4.0 (0.7)	4 (0)	2.4 (1.1)	2 (1)	2.7 (0.8)	3 (1)	4.2 (1.0)	4 (1)	13.2	13
	Hand- wrist complaints	An X-ray is not recommended to diagnose osteoarthritis of the hand or wrist	4.1 (0.8)	4 (1)	3.0 (0.9)	3 (2)	2.4 (0.7)	2 (1)	4.3 (0.8)	4 (1)	13.8	13
	Lumbar radicular syndrom	Ordering imaging (X-ray, CT- or MRI-scan) for patients with a lumbar radicular syndrome is not recommended	4.2 (0.6)	4 (1)	2.4 (1.0)	2 (1)	2.6 (0.8)	3 (1)	4.4 (1.0)	5 (1)	13.5	14
		Benzodiazepines are not recommended in patients with non-specific low-back pain	4.37 (0.7)	4 (1)	2.0 (0.9)	2 (1)	2.5 (0.9)	3 (1)	3.5 (1.3)	4 (2)	12.3	13
		Routinely referring to the physiotherapist for treatment of lumbar radicular syndrome is not recommended	4.4 (0.8)	4 (1)	3.3 (1.2)	3 (2)	2.8 (0.9)	3 (1)	4.4 (0.8)	4 (1)	15.0	14
	Non-specific low back pain	Imaging of the lower back in patients with non-specific low back pain is not recommended	4.7 (0.8)	5 (1)	2.6 (0.8)	3 (1)	2.4 (0.7)	2 (1)	4.5 (0.7)	5 (1)	14.2	15
		Benzodiazepines are not recommended in patients with nonspecific low back pain	4.4 (0.9)	4 (1)	1.8 (0.8)	2 (1)	2.6 (1.0)	3 (2)	3.6 (1.3)	4 (2)	12.5	13
	Non-traumatic knee complaints	Imaging (X-ray or MRI-scan) to determine or rule out osteoarthritis of the knee, chondromalacia patellae, patellar tendinitis, prepatellaris bursitis, iliotibial band syndrome, and Osgood-Schlatter disease is not recommended	4.4 (0.9)	4 (1)	3.0 (1.1)	3 (2)	2.3 (0.7)	2 (1)	4.2 (1.1)	5 (1)	13.8	14
		Non-medical treatment such as taping, arch support, or shockwave are not recommended in patients with complaints caused by chondromalacia patellae, patellar tendinitis, prepatellaris bursitis, iliotibial band syndrome, and Osgood-Schlatter disease	3.8 (0.7)	4 (1)	1.8 (0.7)	2 (1)	2.5 (0.8)	3 (1)	3.9 (1.1)	4 (2)	12.0	13
		Referral to an orthopedic surgeon or sports physician for prepatellar bursitis, patellofemoral pain syndrome, jumper's knee or Osgood-Schlatter's disease is not recommended	3.8 (0.7)	4 (1)	2.1 (0.)	2 (1)	2.7 (1.0)	3 (1)	4.5 (1.0)	5 (1)	13.1	14
		Intra-articular injections with hyaluronic acid is not recommended as treatment for patients with knee osteoarthritis	3.9 (1.,0)	4 (1)	1.2 (0.6)	1 (0)	2,8 (1.0)	3 (1)	4,2 (1.0)	5 (1)	12	13
**Other**	Pain	Do not give a repeat prescription for opioids without a consultation first	3.8 (0.9)	4 (1)	3.8 (1.0)	4 (1)	2.9 (0.9)	3 (2)	3.9 (1.3)	4 (2)	14.3	15
	Arthritis	Laboratory blood tests (C-reactive protein and ESR) are not indicated if the GP is suspicious of arthritis	3.5 (0.8)	4 (1)	3.1 (1.1)	3 (2)	2.3 (0.7)	2 (1)	3.7 (0.8)	4 (1)	12.5	13
	Medically unexplained symptoms	Referring to a monodisciplinary pain specialist is not recommended if the treatment consists of only local invasive pain control such as denervation, and injections with analgesics, corticosteroids, or a sclerosing drug	3.6 (1.0)	4 (1)	2.3 (1.0)	2 (1)	3.1 (0.9)	3 (1)	4.2 (1.1)	5 (1)	13.1	14
	Warts	Do not treat warts longer than 3 months with cryotherapy	4.4 (1.00)	4 (1)	2.2 (0.9)	2 (1)	2.72 (0.8)	3 (1)	3.5 (1.1)	4 (1)	12.8	13

*SD* Standard deviation, *IR* Interquartile range

Concerning the prevalence of the low-value care practice, high scoring recommendations were refill prescription of opiates (60% of the GPs prescribed them frequently to very often), and antibiotics for an infectious conjunctivitis due to a banal pathogen (51% of the GPs prescribed them frequently to very often). Concerning the prevalence of symptoms/clinical picture, the recommendations with the highest median score of 5 were imaging in patients with non-specific low-back pain, oral antibiotics in children with acute otitis media, and decongestive nasal spray as treatment for acute otitis media in children. Ten recommendations had the highest median score of 5 on the potential for cost reduction (criterium 4), with the highest scored recommendation imaging of low-back pain (89% indicated somewhat/fully agree on the potential for cost reduction).

## Discussion

We identified 385 do-not-do recommendations in 92 Dutch general practice guidelines. Considering the prevalence, potential harm to patients, and cost reduction, 30 do-not-do recommendations with highest priority for de-implementation activities were selected. This list can be used to increase awareness of low-value care among GPs and for de-implementation strategies.

Some of our do-not-do recommendations were also included in the lists of other countries as part of the Choosing Wisely campaign or comparable initiatives. The most mentioned recommendation is not ordering imaging in patients with non-specific low back pain without red flags [12, 25–27]. Another common recommendation is not prescribing benzodiazepines for several indications [25, 28, 29]. In our list the recommendation against the use of benzodiazepines was selected for low-back pain problems. Four recommendations on our list concern antibiotics prescription. Although antimicrobial resistance and antimicrobial use in the Netherlands are among the lowest in the world, [30, 31] GPs noticed that unnecessary prescription of antibiotics remains an important topic.

In a recent study of Kool et al. in the Netherlands, showed a wide practice variation of ordering an X-ray [32]. In order to reduce this low-value care service, A survey on primary care clinicians’ perspectives on reducing low-value care showed that GPs need more time for a good explanation to the patient, and education for both the GPs and other healthcare providers as well as patients [18]. These findings confirm the topics that have been identified in our study and that further de-implementation activities are needed. Successful examples of de-implementation strategies for reducing imaging for patients with low-back pain exist, and they focus on both clinicians and patients [33, 34].

### Strengths and limitations

A strength of this study was the systematic identification of recommendations from all recent national guidelines, specifically for GPs. Therefore, the list covers a broad scope of conditions seen in GP care practice. We asked GPs to score the relevance for GP practice so the list is also a good reflection of common practice based on the guideline recommendation. This is a crucial condition for actual de-implementation, as the guidelines are integrated in national quality policy and implemented using multiple strategies such as in CME, audit and feedback, patient education, and practice accreditation [35]. There are several limitations of the methodology of our study. Firstly, we did not assess the evidence for each recommendation, nor did we quantify the prevalence and actual cost. Secondly, all guidelines were screened based on the exact formulation in guideline, which might be arbitrary in some cases. For example: “Strive to prescribe opioids for the shortest duration of time.” is a positive formulated recommendation, whereas “Do not prescribe opiates longer than needed.” is formulated as a do-not-do recommendation. We therefore might have missed low-value care practices that were not specifically formulated as a do-not-do recommendations. Finally, for prioritizing we had an average response rate of 8.8% and therefore the results may be influenced by non-response bias. On the other hand, the absolute number of respondents is acceptable and similar as in other surveys among GPs conducted on a national level. Moreover, the respondents were representative concerning age and location of the practices across the Netherlands.

### Implications for research and practice

Changing behavior in clinical practice is difficult due to several barriers on the level of the health care provider, the patient, social context, and the organizational and political context [36]. Patients often expect diagnostic certainty and hope for treatment interventions. This may be due to fear of serious illness and lack of knowledge [37, 38]. Other barriers to reducing low-value care practices are GPs time constraints, community standards, lack of tools and communications skills to support shared decision making, and fear for being sued [11, 18, 19, 37]. Increased cost-consciousness and awareness of low-value care among GPs is associated with providing less low-value practices [19]. The development of a list of low-value care services is the first step in reducing these services in clinical practice. The de-implementation strategies should be tailored to the identified local barriers and facilitators as for each recommendation the influencing factors are different in the local context. The selected do-not-do recommendations can be used by GPs and policy makers worldwide, as they are supported with the latest evidence from international studies. Although these recommendations are probably not formulated in each international guideline, the list can be used to create awareness on low-value care and for designing strategies to reduce low-value care in other countries as well. Future qualitative research with GPs on their thoughts about this list could contribute to further validity of the list. Future research could focus on the volume and variation of these low-value care practices, the barriers to reducing the low-value care, and what GPs need to change their practice. Routine monitoring with administrative data with sufficient clinical detail to assess appropriateness of care and risk adjustment is necessary to estimate the magnitude of the problem and the potential cost reduction. The developed list with do-not-do recommendations with a high priority for de-implementation activities according to the GPs could be used as a starting point for measurement of low-value care practices, and to raise awareness on low-value care amongst GPs [35, 39]. This routine monitoring should primarily facilitate GPs to learn about their own practice and stimulate them to improve. In addition, concrete plans on how to de-implement this low-value care are needed to change practice. For a structural change systematic, repeated and purposeful efforts are required [40]. For further implementation research we will collaborate with the Dutch College of General Practitioners to start de-implementation projects based on this list, starting with the assessment of some of these low-value care practices. It is important to disseminate the list with a targeted communication campaign in order to raise awareness amongst GPs and patients. Engaging patient organizations and patients in de-implementation activities, e.g. through educational materials or tools for shared decision making, could help to reduce low-value care [41].

## Conclusion

Dutch clinical guidelines include many do-not-do recommendations that are perceived as highly relevant by the GPs. The list of 30 do-not-do recommendations identified by GPs can be used to raise awareness of low-value care among all GPs. As the recommendations are supported with the latest evidence from international studies, primary healthcare professionals and policy makers worldwide can use the list for further validating the list in their local context and designing strategies to reduce low-value care.

## Data Availability

The datasets generated and analysed during the current study are not publicly available because survey respondents were not informed about data sharing and therefore did not give consent for this by completing the survey. This was not yet customary at the time of this study. Data are available from the corresponding author on reasonable request.
